# Supplementary material for: An integrated single-cell RNA-seq map of human neuroblastoma tumors and preclinical models uncovers divergent mesenchymal-like gene expression programs
Source: Genome Biol. 2024 Jun 19;25:161. doi: 10.1186/s13059-024-03309-4 (PMC11186099; doi:10.1186/s13059-024-03309-4)
Supplement: Supplementary file 10 — Additional file 10. Review history. [file 13059_2024_3309_MOESM10_ESM.docx]

Review History

**First round of review**

**Reviewer 1**

**Were you able to assess all statistics in the manuscript, including the appropriateness of statistical tests used?**

Yes, and I have assessed the statistics in my report.

**Comments to author:**

The manuscript "An integrated single-cell RNA-seq map of human neuroblastoma tumors and preclinical models uncovers divergent mesenchymal-like gene expression programs" aimed to dissect the mesenchymal phenotypes of NB tumor cells, which has been widely described in previous cell line studies. The authors generated single-cell RNA-seq maps of neuroblastoma cell lines, patient-derived xenograft models (PDX), and a genetically engineered mouse model (GEMM). Then, they developed an unsupervised machine learning approach ('automatic consensus nonnegative matrix factorization' (acNMF)) to compare the gene expression programs found in preclinical models to a large cohort of patient tumors. The authors only identified weak mesenchymal-like program in pre-treated clinical tumors, which is different from cell lines for mesenchymal states of tumor cells. Finally, the authors show that the weak-mesenchymal-like program could be induced after chemotherapy. This observation is consistent with some previous studies (PMID: 36306349; PMID: 28650485).
Overall, this study is well presented, providing a clearer description of adrenergic/mesenchymal of NB tumor cells and a resource for the community.

Some major and minor issues are as follow:
1. acNMF was developed and applied for discrete cell types (neuroblastoma cancer cells, immune cells, stromal cells, and endothelial cells). The mesenchymal program they identified actually reflect the transcriptional signature of normal fibroblast (low CNV status) rather than as mesenchymal-like neuroblastoma cells. The authors should also perform acNMF analysis for only tumor cells from human clinical tumors, cell lines and PDXs and identify intrinsic mesenchymal program. In general, NB tumor cells in human clinical samples expressed weak or low levels of mesenchymal signatures. I want to know more transcriptional difference between normal fibroblasts and malignant tumor cells in human clinical samples or cell lines. For example, if you can identify some specific mesenchymal genes that are only expressed in normal fibroblasts but not tumor cells?
2. The authors uncovered three tumor cell specific programs: Adrenergic I, Adrenergic II, and Neuroblastoma-MYC (NB-MYC). Why the authors named the third program as NB-MYC? Is it the MYCN-amp tumor specific program?
3. RNA-velocity was performed, showing adrenergic to mesenchymal transition in drug-treated tumors. Can the author also show RNA-velocity in paired pre-treated tumors and quantify the transitional proportion difference in different conditions?
4. The authors have generated single-cell RNA-seq maps of neuroblastoma cell lines. Could the authors show more results (for example, tumor cell compositions and transition using RNA velocity) in neuroblastoma cell lines.
5. Could the authors performed additional analysis to identify EMT associated TF networks underlying the adrenergic to mesenchymal transition using single-cell data. if mesenchymal related-TFs activities (such as TWIST1 and PRRX1) are enhanced after chemotherapy?
6. Chemotherapy will preferentially kill high proliferative cells. According to recent studies, mesenchymal-like phenotype is enriched after chemotherapy and s acquired resistance to chemotherapy. Can the authors pay attention to the cycling behaviors of adrenergic/mesenchymal subtypes of tumor cells?

**Reviewer 2**

**Were you able to assess all statistics in the manuscript, including the appropriateness of statistical tests used?**

Yes, and I have assessed the statistics in my report.

**Comments to author:**

In this manuscript, Chapple et al. build an integrated single-cell RNA-seq dataset from neuroblastoma tumors, cell lines, patient-derived xenograft models, and a genetically engineered mouse model. The authors developed an unsupervised approach, automatic consensus nonnegative matrix factorization (acNMF), and employed this method to characterize the heterogeneity of gene expression signatures across multiple neuroblastoma models. In particular, the authors claimed that the presumptive mesenchymal-like gene expression program in cell lines, which is suggested to be involved in chemotherapy resistance, is not expressed by cancer cells in vivo; rather, fibroblasts expressed the mesenchymal-like signature in patient tumors. The authors identified another weak mesenchymal-like gene expression program that was chemotherapy induced in cancer cells in cell lines and in vivo.

A highlight of this study is an interesting and accessible methodological advancement (acNMF) for identifying gene expression programs from single-cell RNA-Seq data. The authors also make their datasets available through an online database. The identification of distinct neuroblastoma-specific gene expression programs may be useful to the classification of risk in neuroblastoma patients and their findings are validated with RNA in situ hybridization (RNA-ISH).

Although the study is thorough at characterizing gene expression programs in neuroblastoma, the findings still appear to be conflicting with other recent studies. I therefore have some concerns regarding conceptual and technical aspects of the work as summarized below:

1. The authors claim that the mesenchymal-like gene expression signature expressed in neuroblastoma cell lines is exclusively expressed by fibroblasts in vivo, and the signature is not found in neuroblastoma cells in vivo. However, a recent preprint (https://www.biorxiv.org/content/10.1101/2024.01.07.574538v2.full) stated that they found a "subset of tumors had malignant cells in a mesenchymal cell state with molecular features of Schwann cell precursors". How do the authors reconcile these apparently contradicting results?

2. The contribution of the acNMF method to the manuscript's findings is unclear as it was only benchmarked against simulated data. It would improve the manuscript if the authors would compare their method with an alternative approach or reference another study that used another method with similar datasets that yielded different gene expression programs. This would provide a more comprehensive understanding of the method's impact on the results of this study.

3. The authors describe the annotation process of the first set of gene expression programs from the GOSH dataset, stating that: "Two co-authors (Y.K. and P.G.) blindly and independently annotated these 41 programs" and "The two co-authors assigned qualitatively similar annotations to 37 of the 41 programs". What is the criteria for annotation agreement? A more quantitative and objective approach for annotating these gene expression programs should be considered. It is also essential to clarify if this method of annotation was consistently applied across all dataset annotations.

4. There are numerous examples of ambiguous language throughout the manuscript, while some of the claims do not seem strongly supported by their analyses. For example:

a. "Curiously, though, both programs were also weakly but significantly co-expressed with the mesenchymal fibroblast Program 7 (rs = 0.07 P = 1.4 × 10-33 and rs = 0.06, P = 7.1 × 10-25 respectively), also consistent with a very weak or proto-mesenchymal cell state expressed in otherwise adrenergic cells, also consistent with a very weak or proto-mesenchymal cell state expressed in otherwise adrenergic cells"

On what basis do the correlations of 0.07 or 0.06 support the claim of "weakly" or "very weak" "proto-mesenchymal" states?

b. "Cancer associated fibroblasts subpopulations in the neuroblastoma tumor microenvironment strongly express mesenchymal features, heavily resembling mesenchymal-neuroblastoma cell lines."

c. "Rare high-risk neuroblastoma tumors express a weakly enriched mesenchymal-like signature in vivo that can be recapitulated in PDX."

The authors should extensively revise claims based on ambiguous language, which is pervasive throughout the manuscript, otherwise it compromises the interpretability of their results. For example:

The authors state that "Upon a naïve inspection, this seems to imply that mesenchymal neuroblastoma cells have been identified in these tumors. However, one of these programs was expressed exclusively in fibroblasts of the reference normal adrenal sample (Fig. 3m-n; P = 3.75 × 10-40 for mesenchymal enrichment) and thus cannot be expressed in cancer cells". What do the authors mean by a "naïve inspection"? The fact that mesenchymal features were not clearly observed in malignant cells does not preclude that cells "cannot" express them.

The authors state, "Cancer associated fibroblasts subpopulations in the neuroblastoma tumor microenvironment strongly express mesenchymal features, heavily resembling mesenchymal-neuroblastoma cell lines." or "Rare high-risk neuroblastoma tumors express a weakly enriched mesenchymal-like signature in vivo that can be recapitulated in PDX."

The authors state, "The defining genes of this program were dominated by translation initiation and elongation factors (Supplementary Figs. 3d & 3e),consistent with the undifferentiated pathology and hyper-aggressive growth of high-risk neuroblastoma". Could the authors provide a reference for this statement? It is not clear what is the connection between undifferentiated pathology and the mentioned program.

Minor comments:

Figure 4a is overly complicated. While the in-text description and legend is helpful in deciphering the figure, it remains difficult to ascertain meaning from the overall structure. What exactly does the node size mean? How does a node's position within the graph represent sharedness? I think the authors should also consider other methods of representing the sharedness or isolation of the programs as well as the sub-programs. Additionally, the authors state that there are five patient-specific programs. Where are they on the graph?

The quality of the images in Figure 5j are too small and annotation is lacking. Can the authors expand the sizes or include a supplementary figure with the full size images?

The classifier referenced in Figure 5 should be tested on more cells to draw the conclusion that the mesenchymal-like cells are likely normal cells. The low number of cells limits the statistical analysis, and conclusions made from the classifier should not be definitive.

How does the NB-MYC program change from the initial GOSH dataset to the integrative analysis? In the GOSH dataset, the program is patient-specific. However, the authors found that it expanded in their larger analysis. Do the genes that belong to this program change? Are the genes that are lost or gained meaningful?

**Authors Response**

**Point-by-point responses to the reviewers’ comments:**

**Reviewer #1**

**Reviewer #1 Comment #1**

The manuscript "An integrated single-cell RNA-seq map of human neuroblastoma tumors and preclinical models uncovers divergentin mesenchymal-like gene expression programs" aimed to dissect the mesenchymal phenotypes of NB tumor cells, which has been widely described in previous cell line studies. The authors generated single-cell RNA-seq maps of neuroblastoma cell lines, patient-derived xenograft models (PDX), and a genetically engineered mouse model (GEMM). Then, they developed an unsupervised machine learning approach ('automatic consensus nonnegative matrix factorization' (acNMF)) to compare the gene expression programs found in preclinical models to a large cohort of patient tumors. The authors only identified weak mesenchymal-like program in pre-treated clinical tumors, which is different from cell lines for mesenchymal states of tumor cells. Finally, the authors show that the weak-mesenchymal-like program could be induced after chemotherapy. This observation is consistent with some previous studies (PMID: 36306349; PMID: 28650485).

Overall, this study is well presented, providing a clearer description of adrenergic/mesenchymal of NB tumor cells and a resource for the community.

***Response to Reviewer #1 Comment #1***

*We thank the reviewer for this kind summary and their helpful comments, which have greatly improved the quality of the manuscript.*

**Reviewer #1 Comment #2**

Some major and minor issues are as follow:

1. acNMF was developed and applied for discrete cell types (neuroblastoma cancer cells, immune cells, stromal cells, and endothelial cells). The mesenchymal program they identified actually reflect the transcriptional signature of normal fibroblast (low CNV status) rather than as mesenchymal-like neuroblastoma cells. The authors should also perform acNMF analysis for only tumor cells from human clinical tumors, cell lines and PDXs and identify intrinsic mesenchymal program. In general, NB tumor cells in human clinical samples expressed weak or low levels of mesenchymal signatures.

***Response to Reviewer #1 Comment #2***

*We appreciate the insightful inquiry into the detection of intrinsic cancer-specific mesenchymal gene expression signatures. Attempting to first parse cancer and normal cells is commonly applied in the single-cell RNA-seq literature, and we had indeed previously attempted this analysis. However, we found that in practice this approach uniformly led to a decrease in the ability of acNMF to recover gene expression programs, thus making the results worse.*

*Here, we have provided a use case of this phenomenon by subsetting cancer and normal cells in the GOSH dataset and applying independent runs of acNMF on these parsed cell types (See newly attached Supplementary Figure RS1). Whereas acNMF applied to the entire dataset detects 4 GEPs expressed in cancer cells (MYCN, Adrenergic, Neuronal, and Cell Cycle), which were subsequently identified in many other datasets, only 2 of these GEPs were detected in the cancer-restricted subset (Supplementary Figure RS1 - Panel A). The MYCN program is faithfully recapitulated (right panel), but the second program (left panel) conflates components of both the Cell Cycle GEP and Neuronal GEP. Importantly, the Adrenergic GEP is not detected despite the presence of many adrenergic marker genes in these cells (Supplementary Figure RS1 - Panel B below), and this being an extremely well-established program in the neuroblastoma literature. No additional intrinsic mesenchymal-like program is recovered.*

*The reason for the degradation of signal detection when we split the data is due to a reduction of variation of these programs when artificially homogenizing the dataset. NMF favors a sparse and parts-based representation of non-negative data (Lee and Seung, 1999). By this definition, latent factors in gene expression data are discoverable only if they covary across groups of cells. In these neuroblastoma data, the consequence of removing normal cells is the significant reduction of variation of the Adrenergic GEP, which is quite uniformly expressed. This is highlighted in Supplementary Figure RS1 - Panel C. The pie charts describe the normalized relative contribution of each cancer GEP averaged across all cells in each tumor cluster (defined by the original authors’ annotation). We observed that the Adrenergic GEP is reliably identified across all cells in this cancer-restricted subset. The ability of NMF to identify this adrenergic signal relies on the presence of cells that don’t express this signature (e.g. normal cells as an outgroup). Therefore, despite cancer/normal cells often being separated out in the literature, this strategy has little theoretical basis, and in the context of acNMF leads to a loss in power, rather that improving the results, or identifying otherwise obscured programs.*

**Reviewer #1 Comment #3**

I want to know more transcriptional difference between normal fibroblasts and malignant tumor cells in human clinical samples or cell lines. For example, if you can identify some specific mesenchymal genes that are only expressed in normal fibroblasts but not tumor cells?

***Response to Reviewer #1 Comment #3***

*We agree this is an interesting specific question, and indeed, the answer to this, and a host of additional questions (among many not directly addressed in our paper), are pre-computed and can be explored on our web platform (pscb.stjude.org).*

*For example, first navigate to the “Normal fibroblast” program from the GOSH dataset:* [*https://pscb.stjude.org/pscb/_w_3372d584/GEP_reports/human/GOSH/gepSummaryKnitr_3.html*](https://pscb.stjude.org/pscb/_w_3372d584/GEP_reports/human/GOSH/gepSummaryKnitr_3.html)

*By scrolling to the enrichment for “VanGroningen Mesenchymal Genes” a user can immediately see a list of genes that are overexpressed in both “mesenchymal-like” neuroblastoma cells and normal fibroblasts (genes including PCOLCE2, FBN1, COL5A1, POSTN). The top ranked genes overexpressed in these normal fibroblasts, but not mesenchymal-like neuroblastoma cells include LMX1B, PAQR9 and SCARA5.*

*A similar analysis can be performed across any program by navigating to pscb.stjude.org, clicking the "More” link then “Dataset specific GEPs” and navigating to the program of interest.*

*Note: We computed 307 programs across 10 datasets, so it is not possible to report the results of every analysis given the constraints of a conventional academic manuscript, or even Supplementary Materials. However, we were extremely keen to provide a means for such additional questions to be addressed, which is the motivation for providing the community with our explorable web platform.*

**Reviewer #1 Comment #3**

2. The authors uncovered three tumor cell specific programs: Adrenergic I, Adrenergic II, and Neuroblastoma-MYC (NB-MYC). Why the authors named the third program as NB-MYC? Is it the MYCN-amp tumor specific program?

***Response to Reviewer #1 Comment #3***

*This same program is evident in both c-MYC overexpressing and MYCN amplified tumors. We are using NB-MYC as an umbrella term over c-MYC and MYCN. We felt it was more succinct to name it this way.*

**Reviewer #1 Comment #4**

3. RNA-velocity was performed, showing adrenergic to mesenchymal transition in drug-treated tumors. Can the author also show RNA-velocity in paired pre-treated tumors and quantify the transitional proportion difference in different conditions?

***Response to Reviewer #1 Comment #4***

*The comparison of RNA-velocity in cisplatin vs vehicle treated mice was included in Supplementary Figure 10a, but we agree with the reviewer that further explanation is warranted. Similar to our observations in drug-treated mice, cell state dynamics were indeed also observed in the non-drug treated mice.*

*However, in the non-drug treated mice, the RNA velocity signal is overwhelmingly dominated by cell cycle regulators, but the mesenchymal-like transitions evident in the drug treated mice are entirely absent. While the analysis in non-drug treated mice supports the idea that RNA velocity can accurately capture expected transcriptional fluctuations, we feel that adding these vectors to the main figure subtracts from the clarity and ease of discernment of the main idea of identifying the initiation of a transcriptional response to chemotherapy drugs. However, we agree with the reviewer that these findings are germane to the conclusions, particularly providing support for the validity of the overall approach, and, as such, we have now added a new figure describing these transitions to Supplementary Figures 10e-f, which is referenced from line 524 of the main text. We thank the reviewer for this helpful piece of input.*

**Reviewer #1 Comment #5**

4. The authors have generated single-cell RNA-seq maps of neuroblastoma cell lines. Could the authors show more results (for example, tumor cell compositions and transition using RNA velocity) in neuroblastoma cell lines.

***Response to Reviewer #1 Comment #5***

*We agree with the reviewer that it would be interesting to perform these additional analyses on the cell lines. We have now included this result in Supplementary Figure 10a-d, which is referenced in line 524 of the main text.*

**Reviewer #1 Comment #6**

5. Could the authors performed additional analysis to identify EMT associated TF networks underlying the adrenergic to mesenchymal transition using single-cell data. if mesenchymal related-TFs activities (such as TWIST1 and PRRX1) are enhanced after chemotherapy?

***Response Reviewer #1 Comment #6***

*We agree with the reviewer that mesenchymal related TF-activity following chemotherapy is interesting, and indeed, we had validated the increased activity of TWIST1 by RNA-ISH (Fig. 6o-q, and pseudotime analysis (Supplementary Figure 11c). Interestingly, the activity of every TF on each gene expression program is also pre-computed in our web platform (*[*http://pscb.stjude.org*](http://pscb.stjude.org)*).*

*For example, a user can navigate to “Program 39. Neuroblastoma drug-EMT IV (NB853)” from the GEMM dataset:* [*https://pscb.stjude.org/pscb/_w_3372d584/GEP_reports/preclinical/mouse/gepSummaryKnitr_39.html*](https://pscb.stjude.org/pscb/_w_3372d584/GEP_reports/preclinical/mouse/gepSummaryKnitr_39.html)

*By scrolling to the section “Top Ranked Transcription Factors for this Gene Expression Program”, we can see that the top differentially active transcription factors on this program include CDKN1A, PRRX2 and ASCL4.*

*A similar analysis can be performed across any program by navigating to pscb.stjude.org, clicking the "More” link then “Dataset specific GEPs” and navigating to the program of interest.*

**Reviewer #1 Comment #7**

6. Chemotherapy will preferentially kill high proliferative cells. According to recent studies, mesenchymal-like phenotype is enriched after chemotherapy and s acquired resistance to chemotherapy. Can the authors pay attention to the cycling behaviors of adrenergic/mesenchymal subtypes of tumor cells?

***Response to Reviewer #1 Comment #6***

*Yes, as would be expected, there is evidence for the loss of cell cycle related gene expression in the chemotherapy treated cancer cells. Indeed, the cell cycle is “learned” as a discreet activity program by acNMF in every neuroblastoma dataset. Our web platform then contains precomputed co-expression patterns between all programs in every dataset, which allows a user to investigate the activity of the cell cycle in cells expression various programs across the entire set of 10 datasets.*

*Thus, it is possible to investigate these cell cycle programs in detail, for example for the cisplatin treated mice (Program: 43. Cell cycle):* [*https://pscb.stjude.org/pscb/_w_3372d584/GEP_reports/preclinical/mouse/gepSummaryKnitr_43.html*](https://pscb.stjude.org/pscb/_w_3372d584/GEP_reports/preclinical/mouse/gepSummaryKnitr_43.html)

*By scrolling to the “QQ Plot showing correlations with other Gene Expression Programs in this dataset” section of the report, then clicking the “Interactive QQ Plot” link on the QQ plot, it is possible to see the co-expression pattern of this Cell Cycle program and all other programs in this dataset. Unsurprisingly, the cell cycle program is much more highly active in the “Program 30: Neuroblastoma Adrenergic” (r_s_ = 0.32) than any of the mesenchymal/drug treated programs (highest correlation is against “Program 5: Neuroblastoma drug-EMT II (NB847)”, r_s_ = 0.05)*

*A similar analysis can be performed across any program by navigating to pscb.stjude.org, clicking the "More” link then “Dataset specific GEPs” and navigating to the program of interest.*

*Overall, we thank the reviewer for their helpful comment and suggestions, and we hope we have been able to clarify the remaining points.*

**Reviewer #2 comments**

**Reviewer #2 Comment #1**

In this manuscript, Chapple et al. build an integrated single-cell RNA-seq dataset from neuroblastoma tumors, cell lines, patient-derived xenograft models, and a genetically engineered mouse model. The authors developed an unsupervised approach, automatic consensus nonnegative matrix factorization (acNMF), and employed this method to characterize the heterogeneity of gene expression signatures across multiple neuroblastoma models. In particular, the authors claimed that the presumptive mesenchymal-like gene expression program in cell lines, which is suggested to be involved in chemotherapy resistance, is not expressed by cancer cells in vivo; rather, fibroblasts expressed the mesenchymal-like signature in patient tumors. The authors identified another weak mesenchymal-like gene expression program that was chemotherapy induced in cancer cells in cell lines and in vivo.

A highlight of this study is an interesting and accessible methodological advancement (acNMF) for identifying gene expression programs from single-cell RNA-Seq data. The authors also make their datasets available through an online database. The identification of distinct neuroblastoma-specific gene expression programs may be useful to the classification of risk in neuroblastoma patients and their findings are validated with RNA in situ hybridization (RNA-ISH).

***Response to Reviewer #2 Comment #1***

*We thank the reviewer for the kind comments and helpful summary of our paper.*

**Reviewer #2 Comment #2**

Although the study is thorough at characterizing gene expression programs in neuroblastoma, the findings still appear to be conflicting with other recent studies. I therefore have some concerns regarding conceptual and technical aspects of the work as summarized below:

1. The authors claim that the mesenchymal-like gene expression signature expressed in neuroblastoma cell lines is exclusively expressed by fibroblasts in vivo, and the signature is not found in neuroblastoma cells in vivo. However, a recent preprint (<https://nam11.safelinks.protection.outlook.com/?url=https%3A%2F%2Fwww.biorxiv.org%2Fcontent%2F10.1101%2F2024.01.07.574538v2.full&data=05%7C02%7Cpaul.geeleher%40stjude.org%7C262712e483c24daa3d3d08dc2888d681%7C22340fa892264871b677d3b3e377af72%7C0%7C0%7C638429815409954724%7CUnknown%7CTWFpbGZsb3d8eyJWIjoiMC4wLjAwMDAiLCJQIjoiV2luMzIiLCJBTiI6Ik1haWwiLCJXVCI6Mn0%3D%7C0%7C%7C%7C&sdata=jAN3XcLqeAK5794IhfdobbJ%2FI2Ved%2BntsxzWyDN16lI%3D&reserved=0>) stated that they found a "subset of tumors had malignant cells in a mesenchymal cell state with molecular features of Schwann cell precursors". How do the authors reconcile these apparently contradicting results?

***Response to Reviewer #2 Comment #2***

*Our original Discussion section had stated "First, while the neuroblastoma mesenchymal signature was clearly identifiable in neuroblastoma cell lines, we could not identify strong evidence that a similar program is expressed in cancer cells in human tumors—neither by inferred copy number nor by RNA ISH. Regardless, it is logically impossible to conclude the non-existence of such cells. Indeed, they could be identifiable in other tumors, by profiling larger numbers of cells, or by using more sensitive technologies.".*

*Thus, our manuscript absolutely does not preclude new studies from discovering novel neuroblastoma cell states given new samples/data. Indeed, given the transcriptional diversity we have observed across the 10 datasets we analyzed, it seems virtually certain this will be the case. This does not contradict the notion that the existing studies have misclassified CAFs and have missed the CAF subpopulations, which is also true. The realities of neuroblastoma tumor heterogeneity are nuanced, and these complexities must be carefully articulated if the field is to move forward. The validity of the claims in the recently posted preprint, and how it fits together with the rest of the literature, will be assessed during its peer review.*

*Regardless, we have now cited this new preprint (ref. 41) and attempted to place this paper in the context of our findings, re-working the 3^rd^ paragraph of our Discussion (lines 614-618), and stating specifically of the preprint “Accordingly, a recent large spatial atlas reported evidence of malignant Schwann-like programs in some neuroblastoma tumors^41^, thus, as the available data continues to grow, it is likely that further nuances will continue to be discovered.”.*

**Reviewer #2 Comment #3**

2. The contribution of the acNMF method to the manuscript's findings is unclear as it was only benchmarked against simulated data. It would improve the manuscript if the authors would compare their method with an alternative approach or reference another study that used another method with similar datasets that yielded different gene expression programs. This would provide a more comprehensive understanding of the method's impact on the results of this study.

***Response to Reviewer #2 Comment #3***

*It is not possible to quantitatively benchmark methods for modeling single-cell RNA-seq using conventional datasets, where the ground truth is not known. However, we agree with the reviewer that this aspect of the study could be bolstered and we have now added additional comparisons against popular existing methods within our benchmarking framework.*

*Specifically, we have assessed the performance of iNMF (Gao, Nature Biotechnology, 2021), SignatureAnalyzer (Weiner, Genome Biology, 2019), and the variational autoencoder scVI (Lopez,* *Nature Method, 2021). We found that, unsurprisingly, these methods do not accurately recover ground truth programs in our simulated dataset (iNMF k = 35, ScVI k = 36, SignatureAnalyzer k = 21, ground truth: 14 programs). In contrast, acNMF recovered the 14 ground truth programs with 100% specificity and sensitivity. We have included the results of these benchmarks in Supplementary Figure 1u our Results section (line 113) and provided the technical detail in our Methods (lines 749-765)*

*Additionally, a substantial proportion of the initial submission was dedicated to a detailed comparisons of the results we obtained using acNMF on the GOSH dataset against the results obtained by the original authors. The original GOSH paper, applying the de facto analytical tool Seurat, identified 6 clusters in the data. However, acNMF identified 41 gene expression programs, including a highly detailed landscape of immune cells, novel subpopulations of CAFs (which we subsequently validated by RNA ISH), and 3 divergent adrenergic gene expression programs (replicated in other datasets), none of which were discovered using the de facto tools applied to the same data (Fig. 2).*

**Reviewer #2 Comment #3**

3. The authors describe the annotation process of the first set of gene expression programs from the GOSH dataset, stating that: "Two co-authors (Y.K. and P.G.) blindly and independently annotated these 41 programs" and "The two co-authors assigned qualitatively similar annotations to 37 of the 41 programs". What is the criteria for annotation agreement? A more quantitative and objective approach for annotating these gene expression programs should be considered. It is also essential to clarify if this method of annotation was consistently applied across all dataset annotations.

***Response to Reviewer #2 Comment #3***

*Neuroblastoma is a rare and highly heterogenous tissue type. Many gene expression programs we reproducibly recovered across these datasets have never been reported previously (neuroblastoma CAF subpopulations, different adrenergic populations, weak mesenchymal programs, drug-induced mesenchymal programs, etc.). Strictly quantitative annotation of single-cell RNA-seq requires pattern matching to some existing reference set, which even in well studied tissues is fraught with problems. This is why we invested immense time and effort in developing a data exploration platform that would allow human experts to make new discoveries from the data (*[*http://pscb.stjude.org*](http://pscb.stjude.org)*), rather than relying on quantitative pattern matching to existing data. The determination of agreement from the blinded annotators is qualitative, but in the interest of complete transparency we provided the blinded annotations and notes made by both annotators in Supplementary Table 1, which was the basis for determining agreement and final annotations. Additionally, the annotations of the other datasets were largely based on integrating the blindly annotated GOSH dataset with the other datasets using the graph-based* *representation in Figure 4; this procedure was detailed in Supplementary Tables 2 and 3.*

**Reviewer #2 Comment #4**

4. There are numerous examples of ambiguous language throughout the manuscript, while some of the claims do not seem strongly supported by their analyses.

***Response to Reviewer #2 Comment #4***

*We thank the reviewer for pointing out some aspects of our narrative that were unclear. We tried to clarify these points below, and the revised text should be clearer.*

**Reviewer #2 Comment #4a**

a. "Curiously, though, both programs were also weakly but significantly co-expressed with the mesenchymal fibroblast Program 7 (rs = 0.07 P = 1.4 × 10-33 and rs = 0.06, P = 7.1 × 10-25 respectively), also consistent with a very weak or proto-mesenchymal cell state expressed in otherwise adrenergic cells, also consistent with a very weak or proto-mesenchymal cell state expressed in otherwise adrenergic cells" On what basis do the correlations of 0.07 or 0.06 support the claim of "weakly" or "very weak" "proto-mesenchymal" states?

***Response Reviewer #2 Comment #4a***

*We agree with the reviewer that this specific point is confusing, does not add much, and has been removed.*

**Reviewer #2 Comment #4b**

b. "Cancer associated fibroblasts subpopulations in the neuroblastoma tumor microenvironment strongly express mesenchymal features, heavily resembling mesenchymal-neuroblastoma cell lines."

***Response Reviewer #2 Comment #4b***

*We believe this statement is well supported by the computational analyses, as well as RNA-ISH-based validation presented and we value the opportunity to further clarify this point. Figure 3m-q showed the overexpression of the Van Groningen-defined neuroblastoma mesenchymal marker genes in normal fibroblasts and neuroblastoma CAFs. This result was highly robust and was evident in every in vivo dataset that we analyzed. It is not possible to include details of each of these results from 10 datasets in a conventional academic manuscript (nor Supplement), which is one of the reasons we have provided a web platform where these results can be further explored by a user (*[*http://pscb.stjude.org*](http://pscb.stjude.org)*).*

*Here are links to a partial set of these CAF programs, where a user can scroll to the section “****VanGroningen Mesenchymal Genes****” and see that in every case the classical neuroblastoma mesenchymal marker genes are wildly overexpressed in these CAFs (P-values for the enrichment of the MES signature, which are precomputed in our web platform, are listed below):*

*GOSH dataset:*

- [*https://pscb.stjude.org/pscb/_w_3ad50a5c/GEP_reports/human/GOSH/gepSummaryKnitr_19.html*](https://pscb.stjude.org/pscb/_w_3ad50a5c/GEP_reports/human/GOSH/gepSummaryKnitr_19.html) *(P = 5.6 × 10^-50^)*
- [*https://pscb.stjude.org/pscb/_w_3ad50a5c/GEP_reports/human/GOSH/gepSummaryKnitr_40.html*](https://pscb.stjude.org/pscb/_w_3ad50a5c/GEP_reports/human/GOSH/gepSummaryKnitr_40.html) *(P = 6.99 × 10^-83^)*
- [*https://pscb.stjude.org/pscb/_w_3ad50a5c/GEP_reports/human/GOSH/gepSummaryKnitr_35.html*](https://pscb.stjude.org/pscb/_w_3ad50a5c/GEP_reports/human/GOSH/gepSummaryKnitr_35.html) *(P = 3.4 × 10^-23^)*

*Dong Dataset:*

- [*https://pscb.stjude.org/pscb/_w_3ad50a5c/GEP_reports/human/GSE137804/gepSummaryKnitr_7.html*](https://pscb.stjude.org/pscb/_w_3ad50a5c/GEP_reports/human/GSE137804/gepSummaryKnitr_7.html) *(P = 3.5 × 10^-114^)*
- [*https://pscb.stjude.org/pscb/_w_3ad50a5c/GEP_reports/human/GSE137804/gepSummaryKnitr_6.html*](https://pscb.stjude.org/pscb/_w_3ad50a5c/GEP_reports/human/GSE137804/gepSummaryKnitr_6.html) *(P = 4.2 × 10^-68^)*

*GEMM dataset (****where non-malignant status was validated by RNA ISH (e.g. Fig. 5e-h)****):*

- [*https://pscb.stjude.org/pscb/_w_3ad50a5c/GEP_reports/preclinical/mouse/gepSummaryKnitr_9.html*](https://pscb.stjude.org/pscb/_w_3ad50a5c/GEP_reports/preclinical/mouse/gepSummaryKnitr_9.html) *(P = 3.9 × 10^-49^)*
- [*https://pscb.stjude.org/pscb/_w_3ad50a5c/GEP_reports/preclinical/mouse/gepSummaryKnitr_21.html*](https://pscb.stjude.org/pscb/_w_3ad50a5c/GEP_reports/preclinical/mouse/gepSummaryKnitr_21.html) *(P = 1.4 × 10^-115^)*

**Reviewer #2 Comment #4c**

c. "Rare high-risk neuroblastoma tumors express a weakly enriched mesenchymal-like signature in vivo that can be recapitulated in PDX."

***Response to Reviewer #2 Comment #4c***

*We agree with the reviewer that this statement also needs to be robustly supported by the data. Fig 5i showed the weak statistical enrichment of the classical neuroblastoma mesenchymal signature in a sample NB14 from the Jansky dataset, and one of our PDXs, which we showed stains positive for the classical mesenchymal marker COL1A1 in an IHC assay (Fig. 5j). The analyses underpinning these discoveries are also precomputed in our web platform, where they can be further explored by a user.*

*These weak mesenchymal programs can be explored below (where P-values are clearly much lower than CAFs, but an enrichment is statistically evident):*

*Jansky (NB14):*

[*https://pscb.stjude.org/pscb/_w_3ad50a5c/GEP_reports/human/Jansky/gepSummaryKnitr_12.html*](https://pscb.stjude.org/pscb/_w_3ad50a5c/GEP_reports/human/Jansky/gepSummaryKnitr_12.html) *(P = 1.7 × 10^-6^, overexpressed genes include COL1A1, POSTN, CARBP2 etc.)*

*PDX dataset (sample SJNBL063820_sn;* ***validated by IHC****)*

[*https://pscb.stjude.org/pscb/_w_3ad50a5c/GEP_reports/preclinical/PDX_Human/gepSummaryKnitr_1.html*](https://pscb.stjude.org/pscb/_w_3ad50a5c/GEP_reports/preclinical/PDX_Human/gepSummaryKnitr_1.html) *(P = 1.6 × 10^-12^, overexpressed genes include COL1A1 (Fig. 5j), MEOX2, PROM1 etc.)*

**Reviewer #2 Comment #4d**

The authors should extensively revise claims based on ambiguous language, which is pervasive throughout the manuscript, otherwise it compromises the interpretability of their results. For example: The authors state that "Upon a naïve inspection, this seems to imply that mesenchymal neuroblastoma cells have been identified in these tumors. However, one of these programs was expressed exclusively in fibroblasts of the reference normal adrenal sample (Fig. 3m-n; P = 3.75 × 10-40 for mesenchymal enrichment) and thus cannot be expressed in cancer cells". What do the authors mean by a "naïve inspection"? The fact that mesenchymal features were not clearly observed in malignant cells does not preclude that cells "cannot" express them.

***Response to Reviewer #2 Comment #4d***

*We agree the term “naive inspection” is ambiguous and confusing, thus we have revised this wording (line 243). Additionally, we had intended to communicate that the specific mesenchymal-like program* *found a normal adrenal gland cannot be expressed in cancer cells, because there are no cancer cells in that normal adrenal sample (this was not intended to imply mesenchymal programs cannot be expressed in cancer cells more generally). We have reworded that so it is clearer, now stating on line 245 “However, one of these programs was expressed exclusively in fibroblasts of the reference normal adrenal sample (Fig. 3m-n; P = 3.75 × 10^-40^ for mesenchymal enrichment) and thus this specific program cannot be expressed in cancer cells.”*

**Reviewer #2 Comment #4f**

The authors state, "The defining genes of this program were dominated by translation initiation and elongation factors (Supplementary Figs. 3d & 3e), consistent with the undifferentiated pathology and hyper-aggressive growth of high-risk neuroblastoma". Could the authors provide a reference for this statement? It is not clear what is the connection between undifferentiated pathology and the mentioned program.

***Response to Reviewer #2 Comment #4f***

*We agree this was confusingly worded and we thank the reviewer for highlighting it. The clause “consistent with the undifferentiated pathology” should have appeared in the prior sentence, based on data shown there that marker genes of differentiating chromaffin and neuronal cell types are expressed lower in high-risk tumors. We have now moved this clause to the prior sentence (line 216). We have shown representative H&E stains of high/intermediate/low risk neuroblastoma tumors in Fig. 3l and cited a review article from the pathology literature, which describes the (very well established) undifferentiated histology of high-risk neuroblastoma, contrasting this with the differentiated features evident in low/intermediate tumors (Kloppel et al., ref. 32) – observations that are consistent with the acNMF-based representation of the single-cell RNA-seq data.*

**Reviewer #2 Minor comments**

**Reviewer #2 Comment #5**

Figure 4a is overly complicated. While the in-text description and legend is helpful in deciphering the figure, it remains difficult to ascertain meaning from the overall structure. What exactly does the node size mean? How does a node's position within the graph represent sharedness? I think the authors should also consider other methods of representing the sharedness or isolation of the programs as well as the sub-programs. Additionally, the authors state that there are five patient-specific programs. Where are they on the graph?

***Response to Reviewer #2 Comment #5***

*We agree with the reviewer that more description of the graph structure is needed. The method used for the graph layout is a force-directed algorithm. Conceptually, nodes are assigned repelling forces while edges act as springs to attract adjacent nodes. This method of graph visualization is designed to highlight subtle relationships between groups. To highlight these aspects of the graph layout, we have modified the main text, figure legend, and methods accordingly (lines 318-320, 355-356, and 775 respectively). Additionally, we did not include singleton (unconnected) nodes on the graph. These are relatively rare and can be reviewed in detail on our web platform (http://pscb.stjude.org) by clicking the “More” link and “Dataset specific GEPs”, where, for each dataset, these programs are listed under “These GEPs don't appear in the network”.*

**Reviewer #2 Comment #6**

The quality of the images in Figure 5j are too small and annotation is lacking. Can the authors expand the sizes or include a supplementary figure with the full size images?

***Response to Reviewer #2 Comment #6***

*We agree with the reviewer that the plots in Fig. 5j were small and as suggested we have added full sized versions of these images to Supplementary Figure 8g (referenced from line 442 in the main text).*

**Reviewer #2 Comment #7**

The classifier referenced in Figure 5 should be tested on more cells to draw the conclusion that the mesenchymal-like cells are likely normal cells. The low number of cells limits the statistical analysis, and conclusions made from the classifier should not be definitive.

***Response to Reviewer #2 Comment #7***

*We agree that the conclusions based on the classifier need to be tempered, and that it is not possible to prove a negative in this context. We have altered the conclusion based on the classifier so it now reads that “these analyses, given the scale of the data currently available, did not provide convincing evidence for subsets of neuroblastoma cells that highly express a mesenchymal-like gene expression program in vivo.” (lines 404-407). In our Discussion we also stated that “it is logically impossible to conclude the non-existence of such cells” (referring to mesenchymal neuroblastoma cells). Thus, we have not used the classifier to conclude the non-existence of these cells, which is not possible. Additionally, the classifier did call some “mesenchymal-like” neuroblastoma cells as cancer cells, but only in the context of the cisplatin-treated mouse, line 512-514 (P < 2.2 × 10^-16^ for NB853, P = 9.5 × 10^-11^ for NB847, Supplementary Figs. 9b-f). We thank the reviewer for helping us clarify these important points.*

**Reviewer #2 Comment #7**

How does the NB-MYC program change from the initial GOSH dataset to the integrative analysis? In the GOSH dataset, the program is patient-specific. However, the authors found that it expanded in their larger analysis. Do the genes that belong to this program change? Are the genes that are lost or gained meaningful?

***Response to Reviewer #2 Comment #7***

*There was only one high risk patient in the GOSH dataset, where we initially identified this program, thus the program is patient-specific in that context. However, a statistically similar program was subsequently recovered in MYC/MYCN amplified patients in other datasets. The characteristics of these programs, and their differences across datasets, can be reviewed in detail on our web platform, with some examples listed below:*

*Dong dataset:*

[*https://pscb.stjude.org/pscb/_w_e4f8fc5c/GEP_reports/human/GSE137804/gepSummaryKnitr_33.html*](https://pscb.stjude.org/pscb/_w_e4f8fc5c/GEP_reports/human/GSE137804/gepSummaryKnitr_33.html)

*GEMM dataset:*

[*https://pscb.stjude.org/pscb/_w_e4f8fc5c/GEP_reports/preclinical/mouse/gepSummaryKnitr_17.html*](https://pscb.stjude.org/pscb/_w_e4f8fc5c/GEP_reports/preclinical/mouse/gepSummaryKnitr_17.html)

*PDX dataset:*

[*https://pscb.stjude.org/pscb/_w_e4f8fc5c/GEP_reports/preclinical/PDX_Human/gepSummaryKnitr_17.html*](https://pscb.stjude.org/pscb/_w_e4f8fc5c/GEP_reports/preclinical/PDX_Human/gepSummaryKnitr_17.html)

*Jansky dataset:*

[*https://pscb.stjude.org/pscb/_w_e4f8fc5c/GEP_reports/human/Jansky/gepSummaryKnitr_18.html*](https://pscb.stjude.org/pscb/_w_e4f8fc5c/GEP_reports/human/Jansky/gepSummaryKnitr_18.html)

*Overall, we thank both reviewers for the positive assessment of our study and their helpful comments which have substantially improved the paper.*

**Second round of review**

**Reviewer 1**

The authors have addressed my concerns well. I would recommend to accept this paper.

**Reviewer 2**

In the revised manuscript, Chapple et al. have improved the manuscript by addressing most of the initial concerns. The authors provided a better assessment of their method, acNMF, by comparing the predicted gene expression programs across different methods on simulated data. The authors also clarified the text, thus improving the interpretation of their results. The authors also acknowledged a recent preprint in their response which helped to put their findings into context, in particular regarding the existence of mesenchymal states in neuroblastoma cells. This appears to be a controversial topic in the field, and their findings highlight the nuances in characterizing neuroblastoma mesenchymal gene expression programs.
Overall, the manuscript is thorough but some aspects of the analyses lack objectivity. For example, in calling some gene expression programs with subjective confidence levels, which therefore raises concerns on the relevance and interpretation of certain GEPs.


Regarding "1. Our original Discussion section had stated "First, while the neuroblastoma
mesenchymal signature was clearly identifiable in neuroblastoma cell lines, we could
not identify strong evidence that a similar program is expressed in cancer cells in
human tumors—neither by inferred copy number nor by RNA ISH. Regardless, it is
logically impossible to conclude the non-existence of such cells. Indeed, they could be
identifiable in other tumors, by profiling larger numbers of cells, or by using more
sensitive technologies.". Thus, our manuscript absolutely does not preclude new studies from discovering novel neuroblastoma cell states given new samples/data. Indeed, given the transcriptional diversity we have observed across the 10 datasets we analyzed, it seems virtually
certain this will be the case. This does not contradict the notion that the existing studies
have misclassified CAFs and have missed the CAF subpopulations, which is also true. The realities of neuroblastoma tumor heterogeneity are nuanced, and these complexities must be carefully articulated if the field is to move forward. The validity of the claims in the recently posted preprint, and how it fits together with the rest of the literature, will be assessed during its peer review. Regardless, we have now cited this new preprint (ref. 41) and attempted to place this paper in the context of our findings, re-working the 3rd paragraph of our Discussion (lines 614-618), and stating specifically of the preprint "Accordingly, a recent large spatial atlas reported evidence of malignant Schwann-like programs in some neuroblastoma tumors41, thus, as the available data continues to grow, it is likely that further nuances will continue to be discovered."

The authors write in lines 615, "Accordingly, a recent large spatial atlas reported evidence of malignant Schwann-like programs in some neuroblastoma tumors". It is not clear if the authors are referring to the claim from the atlas preprint that malignant Schwann-like cells were found to exist in this mesenchymal-like state. The authors should state more explicitly that the Schwann-like cells were found in the mesenchymal states.

In the following paragraph of the discussion the authors relate their findings with the "embryonic-diapause" phenotype of drug tolerant cells in breast cancer. The diapause state is primarily characterized by a pronounced reduction in biosynthesis activity (reduced activity of protein synthesis, gene expression, proliferation, mitochondrial activity). The connection between the diapause state and EMT/mesenchymal-like program is not directly addressed in the cited manuscript (PMID: 33417832). Therefore, their interpretation regarding the loss of MYC activity and EMT state with the diapause program may not be supported by the current analyses. The authors should consider removing the statement.

Regarding: "Response to Reviewer #2 Comment #3 It is not possible to quantitatively benchmark methods for modeling single-cell RNA-seq using conventional datasets, where the ground truth is not known. However, we agree with the reviewer that this aspect of the study could be bolstered and we have now added additional comparisons against popular existing methods within our benchmarking framework. Specifically, we have assessed the performance of iNMF (Gao, Nature Biotechnology, 2021), SignatureAnalyzer (Weiner, Genome Biology, 2019), and the variational autoencoder scVI (Lopez, Nature Method, 2021). We found that, unsurprisingly, these methods do not accurately recover ground truth programs in our simulated dataset (iNMF k = 35, ScVI k = 36, SignatureAnalyzer k = 21, ground truth: 14 programs). In contrast, acNMF recovered the 14 ground truth programs with 100% specificity and sensitivity. We have included the results of these benchmarks in Supplementary Figure1u our Results section (line 113) and provided the technical detail in our Methods (lines 749-765) Additionally, a substantial proportion of the initial submission was dedicated to a detailed comparisons of the results we obtained using acNMF on the GOSH dataset against the results obtained by the original authors. The original GOSH paper, applying the de facto analytical tool Seurat, identified 6 clusters in the data. However, acNMF identified 41 gene expression programs, including a highly detailed landscape of immune cells, novel subpopulations of CAFs (which we subsequently validated by RNA ISH), and 3 divergent adrenergic gene expression programs (replicated in other datasets), none of which were discovered using the de facto tools applied to the same data (Fig. 2)."

The benchmarking of acNMF against other methods is a valuable addition that adds to the robustness of the methodology, which could be better highlighted in the main text. I understand that the major goal is comparing with the GOSH paper, however, their 6 clusters are driven by cell type, whereas acNMF detects gene expression programs. It is not necessarily a direct comparison as Seurat clustering is not intended to resolve gene expression programs. While simulated data enables ground truth comparisons, once the accuracy is established, it is still important to assess the gene expression programs found by other methods in the real data in comparison with those found by acNMF. This will improve the biological novelty found using the acNMF method. For instance, some of the programs annotated are only present in very few cells (CAF:Myofibroblastic (POSTN+), Adrenocortical Cells). Clarifying the biological novelty uncovered by this method through comparisons to other methods will bolster the biological relevance of these results.


Regarding "Response to Reviewer #2 Comment #3 Neuroblastoma is a rare and highly heterogenous tissue type. Many gene expression programs we reproducibly recovered across these datasets have never been reported previously (neuroblastoma CAF subpopulations, different adrenergic populations, weak mesenchymal programs, drug-induced mesenchymal programs, etc.). Strictly quantitative annotation of single-cell RNA-seq requires pattern matching to some existing reference set, which even in well studied tissues is fraught with problems. This is why we invested immense time and effort in developing a data exploration platform that would allow human experts to make new discoveries from the data (http://pscb.stjude.org), rather than relying on quantitative pattern matching to existing data. The determination of agreement from the blinded annotators is qualitative, but in the interest of complete transparency we provided the blinded annotations and notes made by both annotators in Supplementary Table 1, which was the basis for determining agreement and final annotations. Additionally, the annotations of the other datasets were largely based on integrating the blindly annotated GOSH dataset with the other datasets using the graph-based representation in Figure 4; this procedure was detailed in Supplementary Tables 2 and 3."

Annotating single-cell data does indeed require a degree of subjectivity. However, it is still difficult to grasp the biological meaning behind the annotation agreement status or confidence based on "subjective assignment of confidence levels" (line 785). This is especially relevant when programs are annotated based on just a few cells such as the inflammatory cytokine/signaling or adrenocortical cells programs (Figure 2). The authors could provide a better description on the programs that have high levels of confidence and are the focus of the downstream analyses.

Regarding "Response Reviewer #2 Comment #4a. We agree with the reviewer that this specific point is confusing, does not add much, and has been removed."

It seems that the updated manuscript still contains the referred text (see lines 447 to 450). The authors should revisit this change.

Regarding "We believe this statement is well supported by the computational analyses, as well as RNA-ISH-based validation presented and we value the opportunity to further clarify this point. Figure 3m-q showed the overexpression of the Van Groningen-defined neuroblastoma mesenchymal marker genes in normal fibroblasts and neuroblastoma CAFs. This result was highly robust and was evident in every in vivo dataset that we analyzed. It is not possible to include details of each of these results from 10 datasets in a conventional academic manuscript (nor Supplement), which is one of the reasons we have provided a web platform where these results can be further explored by a user (http://pscb.stjude.org).
Here are links to a partial set of these CAF programs, where a user can scroll to the section "VanGroningen Mesenchymal Genes" and see that in every case the classical neuroblastoma mesenchymal marker genes are wildly overexpressed in these CAFs (P-values for the enrichment of the MES signature, which are precomputed in our web platform, are listed below):"

The description provided in the response is helpful in justifying their claim. I suggest the authors better articulate such evidence in the main text.

Regarding "Response to Reviewer #2 Comment #4c We agree with the reviewer that this statement also needs to be robustly supported by the data. Fig 5i showed the weak statistical enrichment of the classical neuroblastoma mesenchymal signature in a sample NB14 from the Jansky dataset, and one of our PDXs, which we showed stains positive for the classical mesenchymal marker COL1A1 in an IHC assay (Fig. 5j). The analyses underpinning these discoveries are also precomputed in our web platform, where they can be further explored by a user. These weak mesenchymal programs can be explored below (where P-values are clearly much lower than CAFs, but an enrichment is statistically evident):"

The authors have addressed the concern.

Regarding "Response to Reviewer #2 Comment #4d We agree the term "naive inspection" is ambiguous and confusing, thus we have revised this wording (line 243). Additionally, we had intended to communicate that the specific mesenchymal-like program found a normal adrenal gland cannot be expressed in cancer cells, because there are no cancer cells in that normal adrenal sample (this was not intended to imply mesenchymal programs cannot be expressed in cancer cells more generally). We have reworded that so it is clearer, now stating on line 245 "However, one of these programs was expressed exclusively in fibroblasts of the reference normal adrenal sample (Fig. 3m-n; P = 3.75 × 10-40 for mesenchymal enrichment) and thus this specific program cannot be expressed in cancer cells." "

The issue with wording was addressed properly.

Regarding "We agree this was confusingly worded and we thank the reviewer for highlighting it. The clause "consistent with the undifferentiated pathology" should have appeared in the prior sentence, based on data shown there that marker genes of differentiating chromaffin and neuronal cell types are expressed lower in high-risk tumors. We have now moved this clause to the prior sentence (line 216). We have shown representative H&E stains of high/intermediate/low risk neuroblastoma tumors in Fig. 3l and cited a review article from the pathology literature, which describes the (very well established) undifferentiated histology of high-risk neuroblastoma, contrasting this with the differentiated features evident in low/intermediate tumors (Kloppel et al., ref. 32) - observations that are consistent with the acNMF-based representation of the single- cell RNA-seq data."

The authors addressed the issue.

Regarding "Response to Reviewer #2 Comment #5 We agree with the reviewer that more description of the graph structure is needed. The method used for the graph layout is a force-directed algorithm. Conceptually, nodes are assigned repelling forces while edges act as springs to attract adjacent nodes. This method of graph visualization is designed to highlight subtle relationships between groups. To highlight these aspects of the graph layout, we have modified the main text, figure legend, and methods accordingly (lines 318-320, 355-356, and 775 respectively). Additionally, we did not include singleton (unconnected) nodes on the graph. These are relatively rare and can be reviewed in detail on our web platform (http://pscb.stjude.org) by clicking the "More" link and "Dataset specific GEPs", where, for each dataset, these programs are listed under "These GEPs don't appear in the network"."

The changes at 355-356 and 775 helped in the understanding of the figure. The authors should write in the text or the figure legend that unconnected nodes are not included in the figure but can be visualized online.

Regarding "Response to Reviewer #2 Comment #6 We agree with the reviewer that the plots in Fig. 5j were small and as suggested we have added full sized versions of these images to Supplementary Figure 8g (referenced from line 442 in the main text)."

The authors addressed this issue properly.

Regarding "Response to Reviewer #2 Comment #7 We agree that the conclusions based on the classifier need to be tempered, and that it is not possible to prove a negative in this context. We have altered the conclusion based on the classifier so it now reads that "these analyses, given the scale of the data currently available, did not provide convincing evidence for subsets of neuroblastoma cells that highly express a mesenchymal-like gene expression program in vivo." (lines 404-407). In our Discussion we also stated that "it is logically impossible to conclude the non-existence of such cells" (referring to mesenchymal neuroblastoma cells). Thus, we have not used the classifier to conclude the non-existence of these cells, which is not possible. Additionally, the classifier did call some "mesenchymal-like" neuroblastoma cells as cancer cells, but only in the context of the cisplatin-treated mouse, line 512-514 (P < 2.2 × 10-16 for NB853, P = 9.5 × 10-11 for NB847, Supplementary Figs. 9b-f). We thank the reviewer for helping us clarify these important points."

The authors better clarified this issue and the change in language is more appropriate given the impossibility of disproving the existence of mesenchymal neuroblastoma cells.

Regarding "Response to Reviewer #2 Comment #7 There was only one high risk patient in the GOSH dataset, where we initially identified this program, thus the program is patient-specific in that context. However, a statistically similar program was subsequently recovered in MYC/MYCN amplified patients in other datasets. The characteristics of these programs, and their differences across datasets, can be reviewed in detail on our web platform, with some examples listed below:"

The authors clarified this point.

**Authors Response**

**Point-by-point responses to the reviewers’ comments:**

Reviewer comment #1

In the revised manuscript, Chapple et al. have improved the manuscript by addressing most of the initial concerns. The authors provided a better assessment of their method, acNMF, by comparing the predicted gene expression programs across different methods on simulated data. The authors also clarified the text, thus improving the interpretation of their results. The authors also acknowledged a recent preprint in their response which helped to put their findings into context, in particular regarding the existence of mesenchymal states in neuroblastoma cells. This appears to be a controversial topic in the field, and their findings highlight the nuances in characterizing neuroblastoma mesenchymal gene expression programs. Overall, the manuscript is thorough but some aspects of the analyses lack objectivity. For example, in calling some gene expression programs with subjective confidence levels, which therefore raises concerns on the relevance and interpretation of certain GEPs.

# *Response to Final Reviewer comment #1*

*We are glad the Final Reviewer's comments are now largely addressed and we thank them for their particularly helpful comments and attention to detail. We have addressed the remaining comments below.*

# Reviewer comment #2

*Regarding* "1. Our original Discussion section had stated "First, while the neuroblastoma mesenchymal signature was clearly identifiable in neuroblastoma cell lines, we could not identify strong evidence that a similar program is expressed in cancer cells in human tumors—neither by inferred copy number nor by RNA ISH. Regardless, it is logically impossible to conclude the non-existence of such cells. Indeed, they could be identifiable in other tumors, by profiling larger numbers of cells, or by using more sensitive technologies.". Thus, our manuscript absolutely does not preclude new studies from discovering novel neuroblastoma cell states given new samples/data. Indeed, given the transcriptional diversity we have observed across the 10 datasets we analyzed, it seems virtually certain this will be the case. This does not contradict the notion that the existing studies have misclassified CAFs and have missed the CAF subpopulations, which is also true. The realities of neuroblastoma tumor heterogeneity are nuanced, and these complexities must be carefully articulated if the field is to move forward. The validity of the claims in the recently posted preprint, and how it fits together with the rest of the literature, will be assessed during its peer review. Regardless, we have now cited this new preprint (ref. 41) and attempted to place this paper in the context of our findings, re-working the 3rd paragraph of our Discussion (lines 614-618), and stating specifically of the preprint "Accordingly, a recent large spatial atlas reported evidence of malignant Schwann-like programs in some neuroblastoma tumors41, thus, as the available data continues to grow, it is likely that further nuances will continue to be discovered."

The authors write in lines 615, "Accordingly, a recent large spatial atlas reported evidence of malignant Schwann-like programs in some neuroblastoma tumors". It is not clear if the authors are referring to the claim from the atlas preprint that malignant Schwann-like cells were found to exist in this mesenchymal-like state. The authors should state more explicitly that the Schwannlike cells were found in the mesenchymal states.

# *Response to Final Reviewer comment #2*

*We agree with the Reviewer, and we have now explicitly stated that the spatial atlas paper describes evidence for “Schwann-like programs (enriched for mesenchymal-like features)”, which provides further clarification (edits on page 35).*

# Reviewer comment #3

In the following paragraph of the discussion the authors relate their findings with the "embryonicdiapause" phenotype of drug tolerant cells in breast cancer. The diapause state is primarily characterized by a pronounced reduction in biosynthesis activity (reduced activity of protein synthesis, gene expression, proliferation, mitochondrial activity). The connection between the diapause state and EMT/mesenchymal-like program is not directly addressed in the cited manuscript (PMID: 33417832). Therefore, their interpretation regarding the loss of MYC activity and EMT state with the diapause program may not be supported by the current analyses. The authors should consider removing the statement.

*Response to Final Reviewer comment #3*

*We agree with the Reviewer, and we have removed this text (page 35-36).*

# Reviewer comment #4

*Regarding*: "Response to Reviewer #2 Comment #3 It is not possible to quantitatively benchmark methods for modeling single-cell RNA-seq using conventional datasets, where the ground truth is not known. However, we agree with the reviewer that this aspect of the study could be bolstered and we have now added additional comparisons against popular existing methods within our benchmarking framework. Specifically, we have assessed the performance of iNMF (Gao, Nature Biotechnology, 2021), SignatureAnalyzer (Weiner, Genome Biology, 2019), and the variational autoencoder scVI (Lopez, Nature Method, 2021). We found that, unsurprisingly, these methods do not accurately recover ground truth programs in our simulated dataset (iNMF k = 35, ScVI k = 36, SignatureAnalyzer k = 21, ground truth: 14 programs). In contrast, acNMF recovered the 14 ground truth programs with 100% specificity and sensitivity. We have included the results of these benchmarks in Supplementary Figure1u our Results section (line 113) and provided the technical detail in our Methods (lines 749-765) Additionally, a substantial proportion of the initial submission was dedicated to a detailed comparisons of the results we obtained using acNMF on the GOSH dataset against the results obtained by the original authors. The original GOSH paper, applying the de facto analytical tool Seurat, identified 6 clusters in the data. However, acNMF identified 41 gene expression programs, including a highly detailed landscape of immune cells, novel subpopulations of CAFs (which we subsequently validated by RNA ISH), and 3 divergent adrenergic gene expression programs (replicated in other datasets), none of which were discovered using the de facto tools applied to the same data (Fig. 2)."

The benchmarking of acNMF against other methods is a valuable addition that adds to the robustness of the methodology, which could be better highlighted in the main text. I understand that the major goal is comparing with the GOSH paper, however, their 6 clusters are driven by cell type, whereas acNMF detects gene expression programs. It is not necessarily a direct comparison as Seurat clustering is not intended to resolve gene expression programs. While simulated data enables ground truth comparisons, once the accuracy is established, it is still important to assess the gene expression programs found by other methods in the real data in comparison with those found by acNMF. This will improve the biological novelty found using the acNMF method. For instance, some of the programs annotated are only present in very few cells (CAF:Myofibroblastic (POSTN+), Adrenocortical Cells). Clarifying the biological novelty uncovered by this method through comparisons to other methods will bolster the biological relevance of these results.

# *Response to Final Reviewer comment #4*

*We thank the reviewer for this excellent suggestion. We agree that additional comparisons in real data will supplement our existing comparison in simulation and comparisons against Seurat (applied by the original GOSH authors). As such, we have now added a comparison of acNMF against the popular method scVI in the GOSH dataset (note: scVI is by far currently the most widely applied method for gene program recovery in single-cell RNA-seq, Lopez et al, Nature Methods (2018), > 1,300 Google Scholar citations (PMID 30504886)). We found that scVI recovered half as many programs compared to acNMF and there is clear evidence that it is missing true gene expression programs: For instance, none of the scVI estimated programs identified endothelial cells (see figure below), which are a known cell type in all tumors (because all tumors are vascularized). We have now provided these comparisons in Supplementary Figure 1v which is referenced in the main text on page 10.*

*As an additional point of clarification, we did not claim that acNMF identifies programs or cell types that could never be identified by any other existing method. Such a claim would not be possible to support. Our principal conclusions were that acNMF can recover ground truth programs in simulation (scVI, SignatureAnalyzer and iNMF cannot do this, nor can any other method we are aware of). Additionally, acNMF can produce a rich and biologically meaningful representation of neuroblastoma datasets, which are consistent across different datasets, single-cell profiling technologies, and even humans/preclinical models. The acNMF results also yield insights that can be validated by orthogonal approaches (e.g. RNA-ISH). These primary claims we believe are well supported by our results.*

# Final Reviewer comment #5

*Regarding* "Response to Reviewer #2 Comment #3 Neuroblastoma is a rare and highly heterogenous tissue type. Many gene expression programs we reproducibly recovered across these datasets have never been reported previously (neuroblastoma CAF subpopulations, different adrenergic populations, weak mesenchymal programs, drug-induced mesenchymal programs, etc.). Strictly quantitative annotation of single-cell RNA-seq requires pattern matching to some existing reference set, which even in well studied tissues is fraught with problems. This is why we invested immense time and effort in developing a data exploration platform that would allow human experts to make new discoveries from the data ([http://pscb.stjude.org)](http://pscb.stjude.org/), rather than relying on quantitative pattern matching to existing data. The determination of agreement from the blinded annotators is qualitative, but in the interest of complete transparency we provided the blinded annotations and notes made by both annotators in Supplementary Table 1, which was the basis for determining agreement and final annotations. Additionally, the annotations of the other datasets were largely based on integrating the blindly annotated GOSH dataset with the other datasets using the graph-based representation in Figure 4; this procedure was detailed in Supplementary Tables 2 and 3."

Annotating single-cell data does indeed require a degree of subjectivity. However, it is still difficult to grasp the biological meaning behind the annotation agreement status or confidence based on "subjective assignment of confidence levels" (line 785). This is especially relevant when programs are annotated based on just a few cells such as the inflammatory cytokine/signaling or adrenocortical cells programs (Figure 2). The authors could provide a better description on the programs that have high levels of confidence and are the focus of the downstream analyses.

# *Response to Final Reviewer comment #5*

*We thank the reviewer for the excellent suggestion and have provided a description of all programs, including those that are the focus of the downstream analysis (the mesenchymal/CAF programs, the various neuroblastoma programs) in Supplementary Tables S1-3. In addition to this, as we had previously mentioned, all programs can be explored in extraordinary detail on our web platform, with a vast array of precomputed annotations and statistics, which can be used to drive future discoveries beyond what we have detailed in our manuscript:* [*https://pscb.stjude.org/*](https://pscb.stjude.org/)

*As a minor point of clarification, the reviewer has correctly pointed out that only a few cells were identified expressing the adrenocortical program. However, this program was reproducibly recovered across 3 different human neuroblastoma datasets, and is an unsurprising cell type in a tumor of the adrenal gland, consistent with the idea that acNMF can robustly recover gene programs, even when cell count is quite low (notably scVI also missed these cells):*

*GOSH:*

[*https://pscb.stjude.org/pscb/_w_b033b79a/GEP_reports/human/GOSH/gepSummaryKnitr_9.ht ml*](https://pscb.stjude.org/pscb/_w_b033b79a/GEP_reports/human/GOSH/gepSummaryKnitr_9.html)

*Dong:* [*https://pscb.stjude.org/pscb/_w_b033b79a/GEP_reports/human/GSE137804/gepSummaryKnitr _9.html*](https://pscb.stjude.org/pscb/_w_b033b79a/GEP_reports/human/GSE137804/gepSummaryKnitr_9.html)

*Kameneva:* [*https://pscb.stjude.org/pscb/_w_b033b79a/GEP_reports/human/Kameneva/gepSummaryKnitr_*](https://pscb.stjude.org/pscb/_w_b033b79a/GEP_reports/human/Kameneva/gepSummaryKnitr_12.html)

[*12.html*](https://pscb.stjude.org/pscb/_w_b033b79a/GEP_reports/human/Kameneva/gepSummaryKnitr_12.html)

Final Reviewer comment #6

Regarding "Response Reviewer #2 Comment #4a. We agree with the reviewer that this specific point is confusing, does not add much, and has been removed."

It seems that the updated manuscript still contains the referred text (see lines 447 to 450). The authors should revisit this change.

*Response to Final Reviewer comment #6*

*We thank the Reviewer for spotting this error, this text has now been removed (page 24).*

# Final Reviewer comment #7

*Regarding* "We believe this statement is well supported by the computational analyses, as well as RNA-ISH-based validation presented and we value the opportunity to further clarify this point. Figure 3m-q showed the overexpression of the Van Groningen-defined neuroblastoma mesenchymal marker genes in normal fibroblasts and neuroblastoma CAFs. This result was highly robust and was evident in every in vivo dataset that we analyzed. It is not possible to include details of each of these results from 10 datasets in a conventional academic manuscript (nor Supplement), which is one of the reasons we have provided a web platform where these results can be further explored by a user ([http://pscb.stjude.org)](http://pscb.stjude.org/).

Here are links to a partial set of these CAF programs, where a user can scroll to the section "VanGroningen Mesenchymal Genes" and see that in every case the classical neuroblastoma mesenchymal marker genes are wildly overexpressed in these CAFs (P-values for the enrichment of the MES signature, which are precomputed in our web platform, are listed below):"

The description provided in the response is helpful in justifying their claim. I suggest the authors better articulate such evidence in the main text.

# *Response to Final Reviewer comment #7*

*We thank the reviewer for the suggestion and have now included additional description in the main text on page 22.*

# Final Reviewer comment #8

*Regarding* "Response to Reviewer #2 Comment #4c We agree with the reviewer that this statement also needs to be robustly supported by the data. Fig 5i showed the weak statistical enrichment of the classical neuroblastoma mesenchymal signature in a sample NB14 from the Jansky dataset, and one of our PDXs, which we showed stains positive for the classical mesenchymal marker COL1A1 in an IHC assay (Fig. 5j). The analyses underpinning these discoveries are also precomputed in our web platform, where they can be further explored by a user. These weak mesenchymal programs can be explored below (where P-values are clearly much lower than CAFs, but an enrichment is statistically evident):"

The authors have addressed the concern.

*Response to Final Reviewer comment #8*

*We thank the Reviewer for the helpful comment, and we are glad we have addressed this.*

# Final Reviewer comment #9

Regarding "Response to Reviewer #2 Comment #4d We agree the term "naive inspection" is ambiguous and confusing, thus we have revised this wording (line 243). Additionally, we had intended to communicate that the specific mesenchymal-like program found a normal adrenal gland cannot be expressed in cancer cells, because there are no cancer cells in that normal adrenal sample (this was not intended to imply mesenchymal programs cannot be expressed in cancer cells more generally). We have reworded that so it is clearer, now stating on line 245 "However, one of these programs was expressed exclusively in fibroblasts of the reference normal adrenal sample (Fig. 3m-n; P = 3.75 × 10-40 for mesenchymal enrichment) and thus this specific program cannot be expressed in cancer cells." "

The issue with wording was addressed properly.

*Response to Final Reviewer comment #9*

*We thank the Reviewer for the helpful comment, and we are glad we have addressed this.*

# Final Reviewer comment #10

*Regarding* "We agree this was confusingly worded and we thank the reviewer for highlighting it. The clause "consistent with the undifferentiated pathology" should have appeared in the prior sentence, based on data shown there that marker genes of differentiating chromaffin and neuronal cell types are expressed lower in high-risk tumors. We have now moved this clause to the prior sentence (line 216). We have shown representative H&E stains of high/intermediate/low risk neuroblastoma tumors in Fig. 3l and cited a review article from the pathology literature, which describes the (very well established) undifferentiated histology of high-risk neuroblastoma, contrasting this with the differentiated features evident in low/intermediate tumors (Kloppel et al., ref. 32) - observations that are consistent with the acNMF-based representation of the single- cell RNA-seq data."

The authors addressed the issue.

*Response to Final Reviewer comment #10 We thank the Reviewer for the helpful comment, and we are glad we have addressed this.*

# Final Reviewer comment #11

*Regarding* "Response to Reviewer #2 Comment #5 We agree with the reviewer that more description of the graph structure is needed. The method used for the graph layout is a forcedirected algorithm. Conceptually, nodes are assigned repelling forces while edges act as springs to attract adjacent nodes. This method of graph visualization is designed to highlight subtle relationships between groups. To highlight these aspects of the graph layout, we have modified the main text, figure legend, and methods accordingly (lines 318-320, 355-356, and 775 respectively). Additionally, we did not include singleton (unconnected) nodes on the graph. These are relatively rare and can be reviewed in detail on our web platform

[(http://pscb.stjude.org)](http://pscb.stjude.org/) by clicking the "More" link and "Dataset specific GEPs", where, for each dataset, these programs are listed under "These GEPs don't appear in the network"."

The changes at 355-356 and 775 helped in the understanding of the figure. The authors should write in the text or the figure legend that unconnected nodes are not included in the figure but can be visualized online.

# *Response to Final Reviewer comment #11*

*We agree with the Reviewer and we have now stated in the figure legend that the unconnected nodes can be explored in our online platform (edits on page 21).*

# Final Reviewer comment #12

*Regarding* "Response to Reviewer #2 Comment #6 We agree with the reviewer that the plots in

Fig. 5j were small and as suggested we have added full sized versions of these images to

Supplementary Figure 8g (referenced from line 442 in the main text)."

The authors addressed this issue properly.

*Response to Final Reviewer comment #12*

*We thank the Reviewer for the helpful comment, and we are glad we have addressed this.*

# Final Reviewer comment #13

*Regarding* "Response to Reviewer #2 Comment #7 We agree that the conclusions based on the classifier need to be tempered, and that it is not possible to prove a negative in this context. We have altered the conclusion based on the classifier so it now reads that "these analyses, given the scale of the data currently available, did not provide convincing evidence for subsets of neuroblastoma cells that highly express a mesenchymal-like gene expression program in vivo." (lines 404-407). In our Discussion we also stated that "it is logically impossible to conclude the non-existence of such cells" (referring to mesenchymal neuroblastoma cells). Thus, we have not used the classifier to conclude the non-existence of these cells, which is not possible. Additionally, the classifier did call some "mesenchymal-like" neuroblastoma cells as cancer cells, but only in the context of the cisplatin-treated mouse, line 512-514 (P < 2.2 × 10-16 for NB853, P = 9.5 × 10-11 for NB847, Supplementary Figs. 9b-f). We thank the reviewer for helping us clarify these important points."

The authors better clarified this issue and the change in language is more appropriate given the impossibility of disproving the existence of mesenchymal neuroblastoma cells.

*Response to Final Reviewer comment #13*

*We thank the Reviewer for the helpful comment, and we are glad we have addressed this.*

# Final Reviewer comment #14

*Regarding* "Response to Reviewer #2 Comment #7 There was only one high risk patient in the GOSH dataset, where we initially identified this program, thus the program is patient-specific in that context. However, a statistically similar program was subsequently recovered in MYC/MYCN amplified patients in other datasets. The characteristics of these programs, and their differences across datasets, can be reviewed in detail on our web platform, with some examples listed below:"

The authors clarified this point.

*Response to Final Reviewer comment #14 We are glad we have addressed this point.*

*Overall, we thank the Final Reviewer for their additional helpful and insightful comments, which we hope we have addressed. Overall, we believe the paper to now be greatly improved, thanks to the constructive input from all Reviewers and we are hopeful that this manuscript can now be accepted for publication, as we believe it contains important insights and innovations both in single-cell transcriptomics and neuroblastoma biology.*

**Third round of review**

**Reviewer 2**

The authors have done a good job in revising the manuscript and the manuscript is now ready for publication.
